# Impact of fibrosis and sympathetic activity on coronary flow reserve in hypertrophiccardiomyopathy

**DOI:** 10.1186/1532-429X-13-S1-P265

**Published:** 2011-02-02

**Authors:** Oliver Gaemperli, Catherine Gebhard, Rory O'Hanlon, Tevfik Ismail, Ricardo Wage, Susan Clark, Paolo Camici, Sanjay Prasad, Ornella Rimoldi

**Affiliations:** 1 CSC MRC Imperial College, London, UK; 2 Royal Brompton Hospital, London, UK; 3 Royal Brompton Hospital, London, UK; 4 Universita' Vita-Salute - San Raffaele, Milano, Italy; 5 C.N.R. Istituto di Bioimmagini e Fisiologia Molecolare, Milano, Italy

## Introduction

In patients with hypertrophic cardiomyopathy (HCM), positron emission tomography (PET) and cardiac magnetic resonance (CMR) are potentially useful tools for risk stratification. Parameters which could be linked to clinical ourcomes are accurate measures of myocardial ischemia and fibrosis.

## Purpose

We sought to measure simultaneously abnormalities in myocardial blood flow (MBF) and cardiac sympathetic activity using PET and investigate their correlation with the patterns of left ventricular (LV) hypertrophy and myocardial fibrosis assessed with CMR.

## Methods

Thirteen patients (pts) with HCM (age 53±8 years) and 12 healthy age-matched controls (52±10 years) underwent PET with ^11^C-hydroxyephedrine (^11^C-HED) and ^15^O-labeled water (^15^O-H_2_O), and delayed enhancement (DE) CMR on a 1.5 T scanner (Siemens Avanto). LGE images were acquired after intravenous gadolinium-diethylenetriamine penta-acetic acid (0.1 mmol/kg) in short-axis planes identical to cine SSFP images with a breath-hold inversion-recovery gradient echo sequence. The amount of DE was quantitatively assessed on short-axis CMR images with MRI-MASS (Medis) the fibrotic boundaries were identified by “full width half maximum” (FWHM) technique, and fibrosis extent was quantified as a percentage of total left ventricular mass. Myocardial presynaptic catecholamine reuptake/turnover was assessed measuring volumes of distribution (Vd) of ^11^C-HED, and MBF was determined from dynamic ^15^O-H_2_O PET scans at rest and during adenosine stress (140 mcg/Kg/min). PET and CMR data were analysed on a co-registered segmental basis.

## Results

The maximal septal thickness in HCM pts was 21±4 mm, LV mass index (LVMI) 89±19 g/m^2^, and ejection fraction 68±9%. None of the patients had known coronary artery disease. Resting MBF was similar in pts and controls (0.9±0.3 vs. 1.1±0.2 mL/min/g). HCM pts had significantly lower stress MBF (2.1±0.8 vs. 3.1±0.5 mL/min/g; p=0.005) and ^11^C-HED_Vd (44±11 vs. 77±29 mL/g; p=0.002) than normal subjects. In HCM pts 104 out of 205 evaluable LV segments showed DE-CMR > 10% ; in these segments stress MBF was significantly lower (2.04±1 vs. 2.22±0.9 mL/min/g; p=0.003). Binary logistic regression showed a significant association of LGE (p=0.019) and ^11^C-HED_Vd (p=0.0003) with a coronary flow reserve < 2.

^11^C-HED_Vd was correlated with stress MBF (p<0.001) and inversely with the amount of myocardial fibrosis on DE-CMR (p<0.001).

## Conclusions

Our preliminary findings suggest that in HCM pts the presence of fibrosis and the reduced presynaptic nor-adrenaline reuptake, which results in increased sympathetic activity could contribute to aggravate the inducible ischemia due to the anatomical abnormality of the intramural coronary arterioles.

